# Chaotic Dynamics of the Fractional-Love Model with an External Environment

**DOI:** 10.3390/e20010053

**Published:** 2018-01-12

**Authors:** Linyun Huang, Youngchul Bae

**Affiliations:** 1 Department of Biomedical and Electronic Engineering, Chonnam National University, Yeosu 59626, Korea; 2 Division of Electrical Electronic Communication and Computer Engineering, Chonnam National University, Yeosu 59626, Korea

**Keywords:** chaotic dynamic, nonlinear system, fractional order, love model, parameter

## Abstract

Based on the fractional order of nonlinear system for love model with a periodic function as an external environment, we analyze the characteristics of the chaotic dynamic. We analyze the relationship between the chaotic dynamic of the fractional order love model with an external environment and the value of fractional order (α, β) when the parameters are fixed. Meanwhile, we also study the relationship between the chaotic dynamic of the fractional order love model with an external environment and the parameters (*a*, *b*, *c*, *d*) when the fractional order of the system is fixed. When the parameters of fractional order love model are fixed, the fractional order (α, β) of fractional order love model system exhibit segmented chaotic states with the different fractional orders of the system. When the fractional order (α = β) of the system is fixed, the system shows the periodic state and the chaotic state as the parameter is changing as a result.

## 1. Introduction

Fractional calculus is a generalization of the integer-order calculus, which shares the same history length as the study of integer-calculus theory. Before 1960, however, the study of fractional order systems rarely attracted the attention of researchers. Until several decades, especially after the discovery of some physical systems to show the fractional order dynamic characteristics, fractional order system research increasingly received high level of attention. Today, the fractional order system has become a hot global research topic in mathematics and engineering.

In recent years, many researchers have proposed various fractional order chaotic systems with the deep research and exploration of chaotic systems, such as the fractional Rössler system [1,2], fractional Chen system [3,4], fractional Liu system [5,6], and fractional Lorenz system [7,8], among others [9,10].

Over the last three decades, many researchers studied chaotic dynamics in numerous fields such as mathematics, physics, chemistry, engineering, and social science [11,12,13,14,15,16]. In particular, the chaotic behaviors of the habits and minds of humans like addiction [17,18], happiness [19,20], and the “love model” [21,22,23] in terms of the social sciences have been studied.

Strogatz [22] and Sprott [21] explained the behavior of linear and nonlinear systems with respect to the love model, and also proposed the love model based on Shakespeare’s *Romeo and Juliet*. Actually, in mathematics, the love model is based on Romeo and Juliet and can be also defined as the Laura and Petrarch model [24,25] and the Adam and Eve model [26]. However, the “Romeo and Juliet” model is commonly used in the study of nonlinear dynamics by researchers. There are many researchers who study the “Romeo and Juliet” love model to deal with the existence of periodic and chaotic motions. For example, Wauer et al. [27] proposed and analyzed the dynamical models of love with time-varying fluctuations. Son and Park [28] proposed the time-delay effect on the love-dynamic model with the Hopf bifurcation and a periodic-doubling bifurcation diagram. Bae [29,30,31,32,33,34,35,36] proposed that the existence of the periodic and chaotic behaviors that are based on the love model of “Romeo and Juliet,” and it only uses the time series and phase portraits with the same and different time delays and an external force to prove periodic and chaotic behaviors.

There are many studies on love using several of models. However, in real life, we know that love is so complex and uncertain thatit is difficult to describe exact and real love status. Until now, most models of love are based on Strogatz and Sprott, who explained the behavior of linear and nonlinear systems of integer-order love model, but we focus on the fractional order love model. Recently, many researchers have proposed a number of fractional order chaotic dynamics because fractional order can reflect the system changes better than integer order. Compared to the integer order, the fractional order can reflect the “memory dependency” of certain dynamic processes to a certain extent, which means that the current state depends on the past state. In the love model, memory dependency will have a great impact on the results because two people depend on their own memory. The fractional order love model is more convincing and closer to real life than integer love model. In order to represent love affairs more precisely between a man and a woman, we need to introduce the fractional order love model instead of integer love model.

In order to produce chaotic behavior in the dynamic system, the dynamic system needs to be three-dimensional with at least one nonlinear term. Therefore, in the love model between a man and a woman, we need to consider the external environment as an external force to make a three-dimensional system. There are many functions that can be used as an external environment. However, in order to make the system closer to real life, we chose the sine wave function because it can represent positive and negative characteristics for the external environment between a man and a woman. In addition, even though the cosine function can display similar results to the sine wave function, in real life, the sine wave function is more suitable than the cosine function because in the real situation, the impact of the external environment is not from the beginning; it is a process from scratch. Therefore, in this paper, instead of a cosine function, we choose the sine wave function as the external environment. In this paper, we focus the fractional order love model with an external environment, including the economical and psychological situation between a man and a woman, advice from their parents, friends, and relatives, and others. To analyze the chaotic dynamic of the present system more effectively, we use the time series, phase portrait, power spectrum, Poincare map, Maximal Lyapunov exponent (MLE), and bifurcation diagram. We analyze the relationship between the chaotic dynamic of the fractional order love model with an external environment and the value of fractional order (α, β) when the parameters are fixed. Further, we also study the relationship between the chaotic dynamic of the fractional order love model with an external environment and the parameters (*a*, *b*, *c*, *d*) when the fractional order of the system is fixed.

## 2. Love Model

Strogatz [20] proposed the love model for “Romeo and Juliet” with the linear-differential of Equation (1)

(1) $$\begin{array}{c} dR/dt=aR+bJ,\\ dJ/dt=cR+dJ \end{array}$$

where, the parameters “*a*” and “*b*” describe Romeo’s feelings, and c and d describe Juliet’s feelings. There are four situations for Romeo’s romantic style, which are based on the parameters and were suggested by Strogatz and his students [20], including “eager beaver” (*a* > 0, *b* > 0), “narcissistic nerd” (*a* > 0, *b* < 0), “cautious lover” (*a* < 0, *b* > 0), and “hermit” (*a* < 0, *b* < 0).

The simple system is the linear system in which the allowable dynamics are limited, so Equation (1) can be rewrite as Equation (2) through the addition of the nonlinear term.

(2) $$\begin{array}{c} dR/dt=aR+bJ\left(1-J\right),\\ dJ/dt=cR\left(1-R\right)+dJ \end{array}$$

Equation (2), however, cannot be used to produce chaotic behavior because the order of Equation (2) is only two-dimensional. To produce chaotic behavior in the dynamic system, it needs to be three-dimensional, with at least one nonlinear term. Equation (3) can be rewrite into a third-order system through the addition of the 5sin (π*t*) as an external force, as follows:

(3) $$\begin{array}{cc} dR/dt & =aR+bJ\left(1-J\right)+5\sin\left(\pi t\right),\\ \; & dJ/dt=cR\left(1-R\right)+dJ \end{array}$$

Bae [30,31] has proposed a love model of “Romeo and Juliet” wherein the sine wave is an existent external force of the periodic and chaotic behaviors. In such a case, the sum of the system order is 3. In the next section, the focus is the fraction-love model of “Romeo and Juliet” for which the sine wave is an external force and the sum of the system order is gradually reduced.

In Equation (3), to decide range of parameters including *a*, *b*, *c*, and *d*, we consider the corresponding matrix *A* as represented Equation (4).

(4) $$A=\left[\begin{array}{c} a\;\;b\\ c\;\;d \end{array}\right]$$

Then we can get characteristic polynomial as Equation (5).

(5) $$\left|\lambda E-A\right|=\left|\begin{array}{c} \lambda -a\;\;\;\;-b\\ -c\;\;\;\;\lambda -d \end{array}\right|=\left(\lambda -a\right)\left(\lambda -d\right)-bc=\lambda^{2}-\lambda \left(a+d\right)+ad-bc=0$$

The discriminant of the Equation (5) is shown in Equation (6).

(6) $$\Delta ={\left(a+d\right)}^{2}-4\left(ad-bc\right)$$

If Equation (5) has real root, Equation (6) is not less than zero (Δ ≥ 0), that is, *ad* < *bc*. Therefore, in order to find the chaotic parameter in the order of integer love model, the standard of setting the parameter is *ad* < *bc*.

## 3. Chaotic Dynamics of the Fractional Order Love Model with the External Environment

Equation (3) can be modified to Equation (7) through the addition of the fractional order

(7) $$\begin{array}{cc} d^{\alpha }R/dt^{\alpha } & =aR+bJ\left(1-J\right)+5\sin(\pi t),\\ \; & d^{\beta }J/dt^{\beta }=cR\left(1-R\right)+dJ \end{array}$$

where α, β are the fractional orders of the system and fractional order α and β means the magnitude of love status of the two individual, Romeo and Juliet.

In the basic mathematical research and engineering applications, there are four definitions for fractional calculus that are most commonly used: Grunwald-Letnikov fractional calculus, Riemann-Liouville fractional calculus, Caputo fractional calculus, and Riesz fractional calculus [37,38]. Among these definitions, fractional calculus based on the Riemann-Liouville definition is usually used for numerical simulation of fractional model. Therefore, in this paper, we used the Riemann-Liouville definition to carry out the numerical simulation. The Riemann-Liouville definition of the fractional derivative of function *x*(*t*) is given as Equation (8).

(8) $$d^{\alpha }x\left(t\right)/dt^{\alpha }=\left(1/\Gamma \left(n-\alpha \right)\right)d^{n}/dt^{n}\int_{0}^{t}x\left(\tau \right)/{\left(t-\tau \right)}^{\alpha -n+1}d\tau$$

where $\Gamma \left(x\right)=\int_{0}^{\infty }e^{-t}t^{x-1}dt$ is Gamma function.

### 3.1. Analysis of the Systemic Dynamics of the Fixed Parameters (a = −1.5, b = −2, and c = d = 1)

In order to set the parameters of the fractional order love model in Equation (7), we may apply a similar method to Equation (4) to Equation (6). However, this standard method, which applies in the order of the integer love model, is not suited for the fractional order love model. In the fractional order system, the Equation (6) can be less than zero (Δ < 0), which means even if there is an imaginary root in Equation (6), the system has the potential to generate chaos. This can be drawn from Table 1. When parameters b, c, d are fixed as *b* = −2, and *c* = *d* = 1, we found out the value of parameter “*a*” that can produce chaotic motion as the different fractional order value. Table 1 shows the value of parameter “*a*” that can produce chaotic motion with a different fractional order.

Parameter “*a*” describes the extent to which Romeo is encouraged by his own feelings. “*a*” < 0 means Romeo retreats form his own feelings, and the value of “*a*” means the degree of Romeo retreats from his own feelings. As the fractional order become smaller, the value of the parameter “*a*,” which can generate chaotic behavior in the system, also becomes smaller. Therefore, in order to analysis the chaotic dynamic of the system, we set parameter “*a*” as *a* = −1.5, therefore, in this section, the parameters are fixed as *a* = −1.5, *b* = −2, and *c* = *d* = 1.

To describe fractional order of love affairs with parameter value, we set of 0 < α = β = *q* ≤ 1. Therefore, Equation (7) can be rewritten as Equation (9).

(9) $$\begin{array}{cc} d^{q}R/dt^{q}= & \left(-1.5\right)R+\left(-2\right)J\left(1-J\right)+5\sin\left(\pi t\right),\\ \; & d^{q}J/dt^{q}=R\left(1-R\right)+J \end{array}$$

The fraction q can be changed from 1 to 0.5 by 0.05 steps to study the chaotic dynamic of the system. In order to prove the chaotic behaviors in the dynamical system, typically, we use the time series, phase portrait, power spectrum, Poincaré map, and MLE. Generally, since each item cannot provide sufficient condition, we have to use all these items to satisfy the necessary condition to prove chaotic or not in the dynamical system.

#### 3.1.1. Time Series

**Figure 1:**
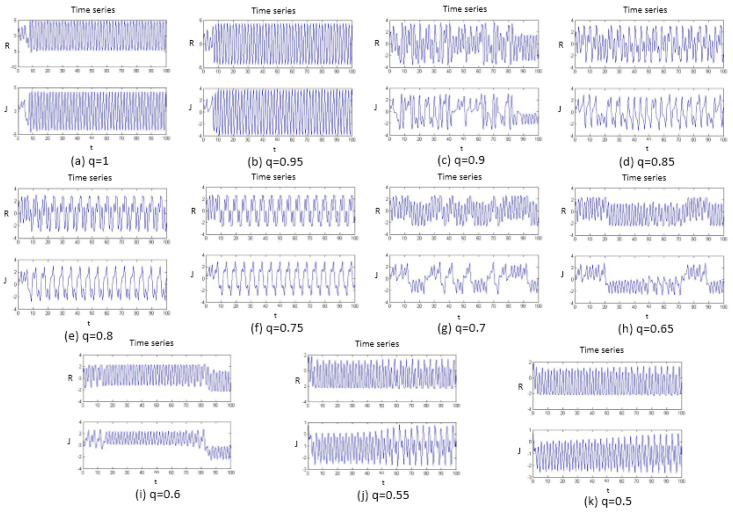
Time series of the system with different fractional order *q* values, and subfigures (**a**–**k**) are the fractional order *q* changed from 1 to 0.5 by 0.05 steps.

The chaotic time series is a definite motion that determines the presence of a system. The chaotic time series is a time series with chaotic-model characteristics. The chaotic time series contains the rich dynamic information of the system. The study of chaos from the time series began with Packard et al. [39]. In terms of the chaotic time series, it is known that the periodic motion corresponds to the rules sequence, and the chaotic motion corresponds to the irregular sequence; therefore, it is possible to intuitively determine whether the system is chaotic by observing the systemic time series. The fraction q can be changed from 1 to 0.5 by 0.05 steps to obtain the time series through the implementation of a computer simulation, as shown in Figure 1.

From Figure 1, when the fraction q is equal to the four situations 1, 0.95, 0.8, and 0.75, the time series shows the regular sequence, while when the fraction q is equal to 0.9, 0.85, 0.8, 0.65, and 0.6, the time series shows the irregular sequence. However, the q situations that are equal to 0.55 and 0.5 are not easy to distinguish. Therefore, the dynamic characteristics of the fractional order love model with the external environment is initially understandable, but to further know the dynamic characteristics of the system, the phase portrait must be observed.

#### 3.1.2. Phase Portrait

**Figure 2:**
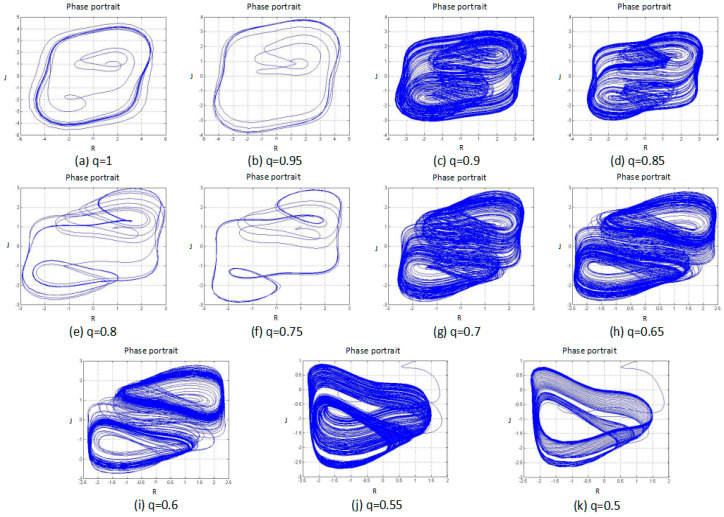
Phase portrait of the system with different fractional order *q* values, and subfigures (**a**–**k**) are the fractional order *q* changed from 1 to 0.5 by 0.05 steps.

A phase portrait is a geometric representation of the trajectories of a dynamical system in the phase plane. Each set of initial conditions are represented by a different curve, or point. According to the direct-observation method, the periodic motion in the phase space corresponds to the closed curve, and the chaotic motion corresponds to the trajectory of the random separation in a certain region. Therefore, by observing the phase portrait of the fractional order love model with the external-force system, it is possible to further determine whether the system is chaotic or not. The results of the systemic phase portrait are shown in Figure 2.

From Figure 2, it is possible to see that when the fraction q is equal to the four situations 1, 0.95, 0.8, and 0.75, the phase-portrait curve is a limit cycle or converges to a single point, which indicates that the system is in a periodic state at these moments. In the remaining cases, the phase-portrait variables exist in a random separation state—that is, chaotic attractors—indicating that, for these cases, the system is in a chaotic state. From the time-series and phase-portrait results, the fractional order system exhibited segmented chaotic states. The observation method here, however, is only a qualitative analysis, so this conclusion needs to be further verified.

#### 3.1.3. Power Spectrum

**Figure 3:**
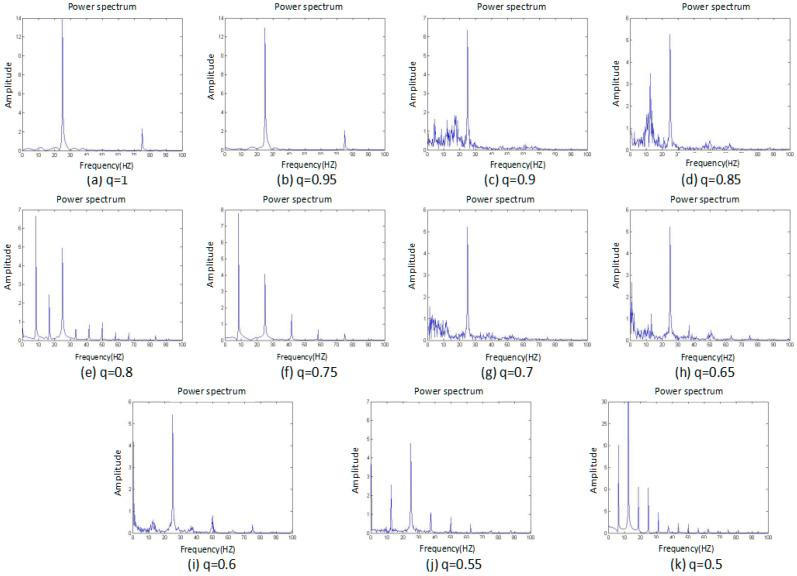
Power spectrum of the system with different fractional order *q* values, and subfigures (**a**–**k**) are the fractional order *q* changed from 1 to 0.5 by 0.05 steps.

The power spectrum of a signal represents the power or more simply the energy of the signal at each frequency. It can also be considered as the range or spectra of energy or power of the given signal derived from the signals’ range of frequencies. Therefore, a power-spectrum analysis can provide the frequency-domain information of the signal. From an analysis of the power spectrum, it is possible to observe whether the systemic characteristics are chaotic or not. For the periodic motion, the power spectrum is a discrete spectrum, while for the chaotic motion, the power spectrum is a continuous spectrum. It is possible to determine whether the system is chaotic by plotting the power spectrum of the system-generated signal. The power spectrum of the system shown in Figure 3.

Figure 3 shows that when the fraction q is equal to the four cases of 1, 0.95, 0.8, and 0.75, the power spectrum is a discrete spectrum. At these moments, the system is in a periodic state. In the remaining situations, the power spectrum is a continuous spectrum, or a chaotic state, so the system exhibits a segmented chaotic state. This conclusion is consistent with the phase-portrait results.

#### 3.1.4. Poincaré Map

Poincaré map is the intersection of a periodic orbit in the state space of a continuous dynamical system with a certain lower-dimensional subspace, called the Poincaré section, transversal to the flow of the system. For the selection of a cross section in a multi-dimensional phase space, this section can be both a plane and a surface. Then, a point series of the continuous dynamic orbit that intersects with the cross section can be considered. From the cut point on the Poincaré map, the motion-characteristics information can be obtained. Different forms of motion pass through the cross section, and the intersectional cross section comprises different distribution characteristics, as follows:

(1) The periodic motion leaves a limited number of discrete points on this cross section;
(2) The quasi-periodic motion leaves a closed curve on the cross section;
(3) The chaotic motion is along a line or a curved-arc distribution point that is set on the cross section.

**Figure 4:**
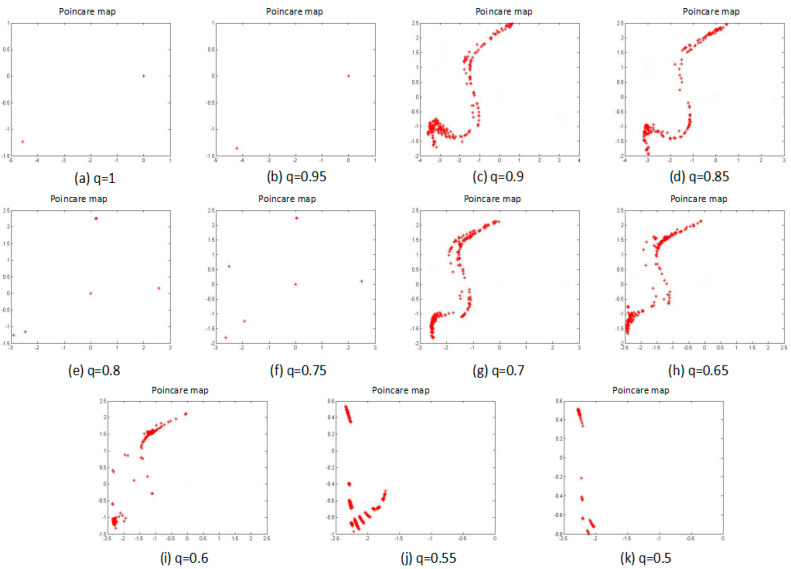
Poincaré map of the system with different fractional order *q* values, and subfigures (**a**–**k**) are the fractional order *q* changed from 1 to 0.5 by 0.05 steps.

Therefore, the points, which are left on the Poincaré map to judge the system status, can be observed. Figure 4 shows the results of the Poincaré map as the fractional order was changed.

From Figure 4, the points when the fraction q is equal to 1, 0.95, 0.8, and 0.75 can be clearly seen, and there is a number of discrete points on the Poincare map that indicate that the system exhibits the periodic motion. The remaining situations are along a line distribution of points on the Poincare map, so the system exhibits the chaotic motion. These results are consistent with the results of the phase portrait and the power spectrum, so until now, the system certainly presents a segmented chaotic state.

So far, all of the methods are used to qualitatively analyze the dynamics of the system. In the next section, the maximal LE of the system is calculated to quantitatively analyze the dynamics of the system.

#### 3.1.5. Maximal Lyapunov Exponent

The definition of the maximal Lyapunov exponent is shown as follows:

(10) $$\lambda =\underset{t\to \infty }{\lim}\;\underset{\delta z_{0\to 0}}{\lim}\frac{1}{t}\ln\frac{\left|\delta z\left(t\right)\right|}{\left|\delta z_{0}\right|}$$

where, lim*δz*_0_→_0_ can make sure of the validity of the linear approximation at any time.

We know the MLE is one of the important dynamic-characteristic measurements of the system and characterizes the average exponential rate of the convergence or the divergence of the system variables in the adjacent phase-space orbits. Especially, the MLE determines whether the adjacent trajectories can move closer to each other to form a stable or unstable point. If the MLE is less than 0, the system shows the periodic motion, while a MLE of more than 0 shows a chaotic systemic motion. Therefore, it is possible to calculate the MLE of the system to quantitatively analyze whether the system is in a chaotic state. Table 2 shows the MLE of the system as different fractional orders when the parameters are fixed.

From Table 1, it is possible to clearly know when the fraction q is equal to 1, 0.95, 0.8, or 0.75, and when the MLE is less than 0, so a periodic-state system can be identified. In the other situations, the MLE is more than 0, so the system is in the chaotic state.

Based on all of the methods that are used, the authors conclude that the state of the fractional order love model with an external environment is related to the fractional order. When the parameters are fixed, the system exhibited the segmented chaotic state with a different fractional order.

### 3.2. Analysis of the Systemic Dynamics of the Fixed Fractional Orders (α = β = 0.85)

**Figure 5:**
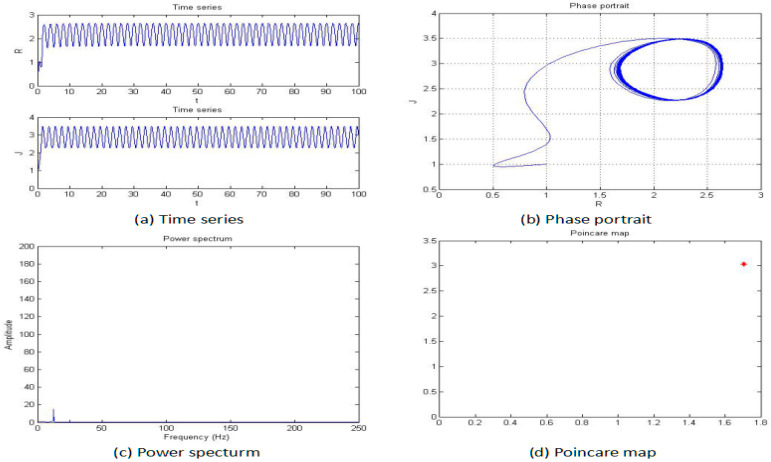
Time series (**a**), phase portrait (**b**), power spectrum (**c**), and Poincaré map (**d**) of the system when *a* = −5.

**Figure 6:**
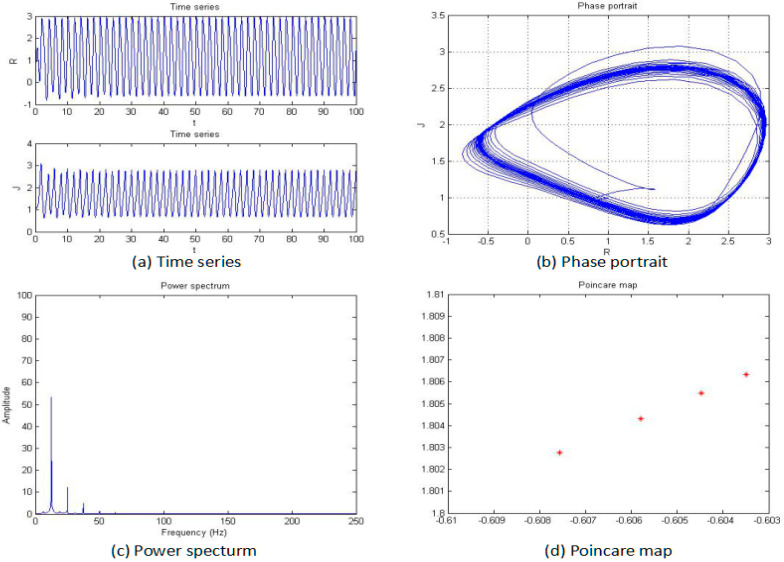
Time series (**a**), phase portrait (**b**), power spectrum (**c**), and Poincaré map (**d**) of the system when *a* = −2.42.

**Figure 7:**
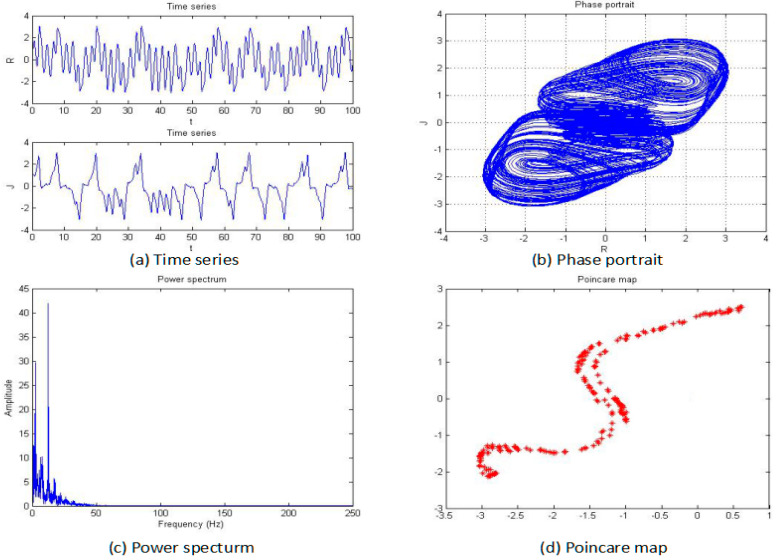
Time series (**a**), phase portrait (**b**), power spectrum (**c**), and Poincaré map (**d**) of the system when *a* = −2.

**Figure 8:**
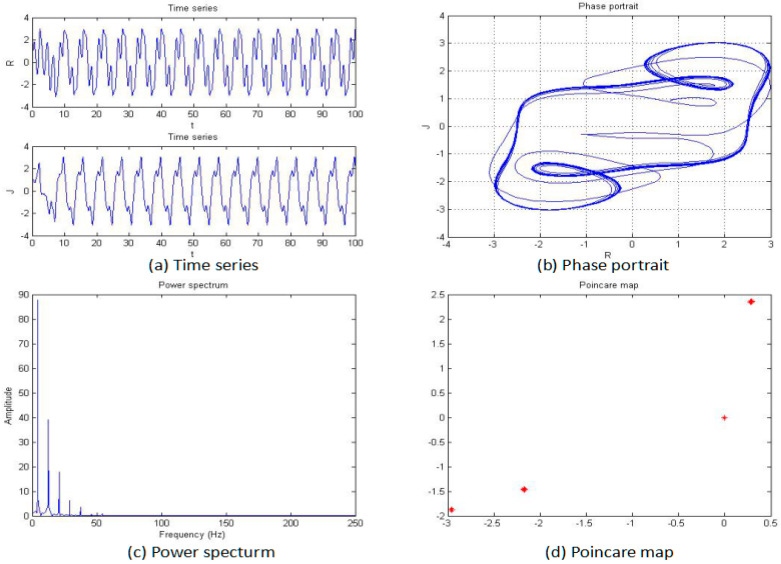
Time series (**a**), phase portrait (**b**), power spectrum (**c**), and Poincaré map (**d**) of the system when *a* = −1.76.

**Figure 9:**
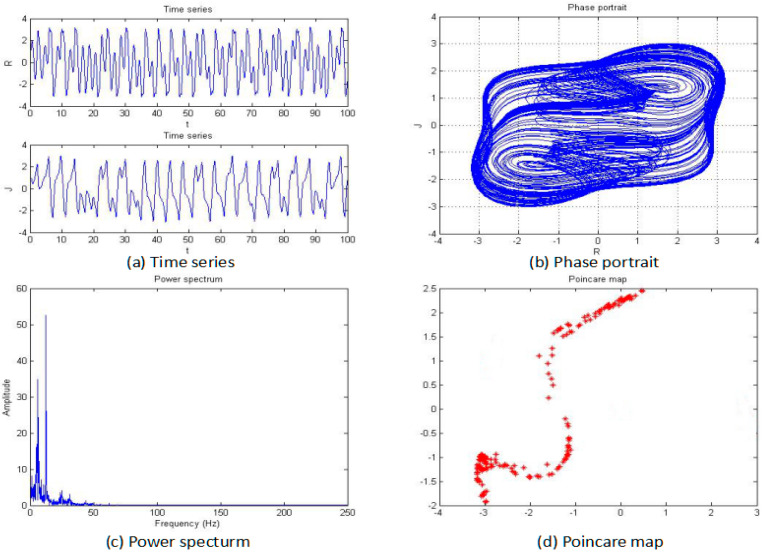
Time series (**a**), phase portrait (**b**), power spectrum (**c**), and Poincaré map (**d**) of the system when *a* = −1.53.

**Figure 10:**
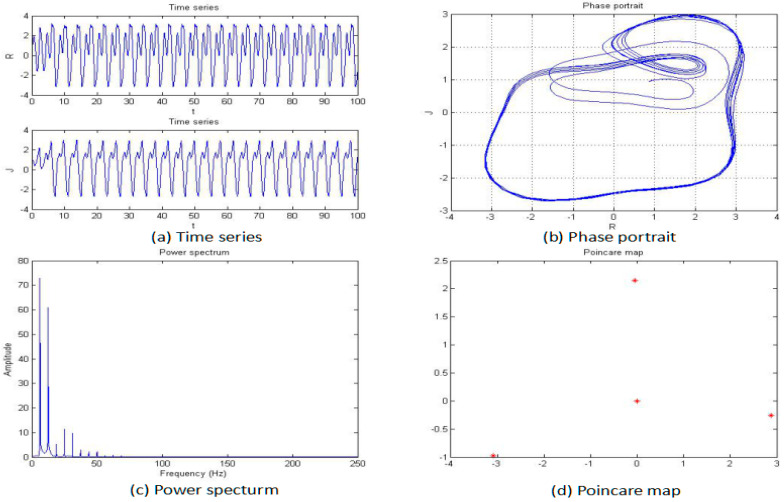
Time series (**a**), phase portrait (**b**), power spectrum (**c**), and Poincaré map (**d**) of the system when *a* = −1.45.

For this section, the fractional order of the system was fixed as 0.85, and the parameters b, c, and d were also fixed as −2, 1, and 1, respectively; then, the parameter “*a*” was changed for an analysis of the chaotic dynamics of the system. In addition, the time series, phase portrait, power spectrum, Poincare map, and MLE used to obtain the results. It is worth mentioning that the bifurcation-diagram method was added for this section to verify the results. Figure 5, Figure 6, Figure 7, Figure 8, Figure 9 and Figure 10 show the time series, phase portrait, power spectrum, and Poincare map of the system when parameter “*a*” is *a* = −5, *a* = −2.42, *a* = −2, *a* = −1.76, *a* = −1.53, and *a* = −1.45, respectively.

**Figure 11:**
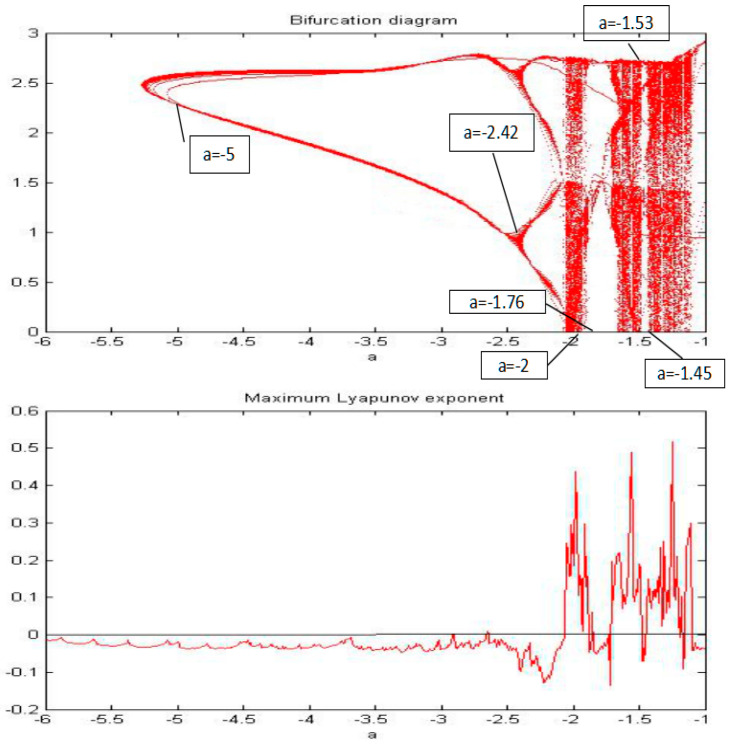
The bifurcation diagram and MLE of the system when parameter “*a*” changed from −6 to −1.

From Figure 5, Figure 6, Figure 7, Figure 8, Figure 9 and Figure 10, it clearly shows when the parameter “*a*” is equal to −2 and −1.53, the system is in the chaotic state, and in the other situations, the system is in the periodic state. Therefore, it is possible to initially conclude that when the fractional order of the system is fixed, the system shows the periodic and chaotic states as the parameter “*a*” is changed. To verify the accuracy of the conclusion, the results of the MLE and the bifurcation diagram are shown in Figure 11.

From the results of the MLE and the bifurcation diagram, it is possible to conclude that when the fractional order of the system is fixed, the system shows the periodic and chaotic states as the parameter is changed.

## 4. Conclusions

In this paper, the time series, phase portrait, power spectrum, Poincaré map, MLE, and bifurcation diagram were used to analyze the characteristics of the chaotic dynamic of the fractional order love model with an external environment. For the analysis, we study the following two aspects of the system. On the one hand, we analyzed the relationship between the chaotic dynamic of the fractional order love model with an external environment and the value of fractional order (α, β) when the parameters are fixed. The results show that the chaotic dynamic of the system is related to the fractional order of the system. When each parameter *a*, *b*, *c*, and *d* is fixed as −1.5, −2, 1, and 1, the fractional order (α = β) changed from 1 to 0.5 by 0.05 steps, and the fractional order love model system exhibited segmented chaotic states. Further, when fractional order (α = β) is equal to 1, 0.95, 0.8, or 0.75, the fractional order love model displayed periodic-state. In the other situations, the system is in the chaotic state. On the other hand, we studied the relationship between the chaotic dynamic of the fractional order love model with an external environment and the parameters (*a*, *b*, *c*, *d*) when the fractional order of the system is fixed. The results show the chaotic dynamic of the system is also related to the parameters of the system. When the fractional order (α = β) of the system is fixed as 0.85, the system exhibited the periodic state and the chaotic state as the parameter is changing. Further, when parameter “*a*” is −5 and −2.42, the fractional order love model exhibited periodic states; when parameter “*a*” is −2 and −1.53, the system is chaotic state. Therefore, the characteristics of the fractional order love model that comprises a sine wave as an external environment are rich and dynamic.

## Figures and Tables

**Table 1:** The value of parameter “*a*” that can produce chaotic motion with different fractional order.

Fractional order α = β	α = β = 1	α = β = 0.9	α = β = 0.8	α = β = 0.7	α = β = 0.6	α = β = 0.5
Parameter “*a*”	*a* = −2.88	*a* = −2.22	*a* = −1.8	*a* = −1.65	*a* = −1.45	*a* = −1.22

**Table 2:** Maximal Lyapunov exponent (MLE) of the system with different fractional orders when the parameters are fixed.

Fractional Order (*q*)	MLE (*λ*)	Dynamic State
*q* = 1	*λ* = −0.0458	periodic
*q* = 0.95	*λ* = −0.0299	periodic
*q* = 0.9	*λ* = 0.0711	chaotic
*q* = 0.85	*λ* = 0.3467	chaotic
*q* = 0.8	*λ* = −0.0331	periodic
*q* = 0.75	*λ* = −0.0344	periodic
*q* = 0.7	*λ* = 0.2456	chaotic
*q* = 0.65	*λ* = 0.3585	chaotic
*q* = 0.6	*λ* = 0.1835	chaotic
*q* = 0.55	*λ* = 0.0754	chaotic
*q* = 0.5	*λ* = 0.0357	chaotic
